# COVID‐19‐related anxiety predicts somatic symptoms in the UK population

**DOI:** 10.1111/bjhp.12430

**Published:** 2020-05-27

**Authors:** Mark Shevlin, Emma Nolan, Marcin Owczarek, Orla McBride, Jamie Murphy, Jilly Gibson Miller, Todd K. Hartman, Liat Levita, Liam Mason, Anton P. Martinez, Ryan McKay, Thomas V. A. Stocks, Kate M. Bennett, Philip Hyland, Richard P. Bentall

**Affiliations:** ^1^ Ulster University Coleraine UK; ^2^ University of Sheffield UK; ^3^ University College London UK; ^4^ Royal Holloway University of London UK; ^5^ Liverpool University UK; ^6^ Maynooth University Ireland

**Keywords:** COVID‐19 pandemic, somatic symptoms, COVID‐19 related anxiety

## Abstract

This study aimed to estimate the association between anxiety associated with COVID‐19 and somatic symptoms, using data from a large, representative sample (*N* = 2,025) of the UK adult population. Results showed that moderate to high levels of anxiety associated with COVID‐19 were significantly associated with general somatic symptoms and in particular with gastrointestinal and fatigue symptoms. This pattern of associations remained significant after controlling for generalized anxiety disorder (GAD), pre‐existing health problems, age, gender, and income. This is the first evidence that anxiety associated with COVID‐19 makes a unique contribution to somatization, above and beyond the effect of GAD.

**Statement of contribution**

***What is already known on this subject?***

Somatic symptoms are commonly reported in the general population, and many are medically unexplained. There is also evidence that high levels of anxiety are associated with reporting more somatic symptoms and increased impairment. Research evidence from previous infectious disease pandemics, such as SARS, showed increased levels of anxiety in the general population during quarantine, and this was associated with increased reporting of somatic symptoms.

***What does this study add?***

During the current COVID‐19 pandemic in the United Kingdom, there may be increased levels of generalized anxiety in the general population, but there may also be anxiety specifically associated with COVID‐19. This study is the first to show that there is a positive association between both generalized anxiety and COVID‐19 specific anxiety and the reporting of somatic symptoms. Indeed, COVID‐19 specific anxiety explained unique variance in somatic symptoms after controlling for generalized anxiety and other potential confounding variables.

It has previously been demonstrated that health pandemics have psychological consequences. Studies of the psychological effects of severe acute respiratory syndrome (SARS), which emerged in 2003, showed that PTSD symptoms, anxiety, and depression were significantly associated with time spent in quarantine (Hawryluck *et al*., 2004). The magnitude of the current quarantine measures imposed by the UK government are much greater and stricter than those imposed in any previous infectious disease pandemic, and negative psychological consequences for the general population are expected. There is recent evidence that the levels of anxiety and depression in the UK population may be elevated compared to pre‐pandemic levels (Shevlin *et al*., 2020), and this is consistent with research findings from other countries (Huang & Zhao, 2020).

To date, one area that has received little attention in the context of the current pandemic is the association between anxiety and somatic symptoms. There is evidence from general population studies that somatic symptoms are frequently reported and positively associated with depression and anxiety (Haug *et al*., 2004; Hinz *et al*., 2017). A recent study in the Hubei province and non‐endemic provinces of China (Yuan *et al*., 2020) found associations between somatic symptoms and levels of anxiety during the first 2 weeks of the COVID‐19 outbreak. As anxiety scores decreased, there was a corresponding decrease in somatic symptoms.

The primary aim of this study was to examine whether self‐reported anxiety related to the COVID‐19 pandemic predicted scores on a validated, multi‐dimensional measure of somatic symptoms in a representative UK adult population sample. In addition, it was predicted that COVID‐19 anxiety would remain significantly associated with somatic symptoms after controlling for multiple confounding variables including age, gender, income, pre‐existing health problems, and generalized anxiety disorder (GAD).

## Methods

### Participants

Data collection started on 23 March 2020, 52 days after the first confirmed COVID‐19 case in the United Kingdom and was completed on 28 March 2020. Participants (*N* = 2,025) were recruited from an online research panel using stratified quota sampling to ensure that the sample characteristics of sex, age, household income (quintiles), and region of the United Kingdom matched the UK population. Subsequent checks ensured that they were also representative of the population in ethnicity, number of people in household, and other important demographic characteristics (for details on recruitment and sample, see McBride *et al*., 2020).

### Measures

Self‐reported gender and age were recorded.

#### Income

Participants were asked ‘Please choose from the following options to indicate your approximate gross (before tax is taken away) household income in 2019 (last year). Include income from partners and other family members living with you and all kinds of earnings including salaries and benefits’; they had to choose one of 5 categories ranging from ‘no income to £15,490 per year’ to ‘£57,931 or more per year’.

#### Pre‐existing health problems

Participants were asked ‘Were you diagnosed with a health condition (e.g., heart or lung disease; diabetes; cancer) before December 31st 2019 (i.e., before the start of the coronavirus COVID‐19 outbreak)?’, and the response options were ‘Yes’ (1) and ‘No’ (0).

#### COVID‐19‐related anxiety

The survey included a question ‘How anxious are you about the coronavirus COVID‐19 pandemic?’; participants were provided with a ‘slider’ (electronic visual analogue scale) to indicate their degree of anxiety with ‘0’ and ‘100’ at the left‐ and right‐hand extremes, respectively, and 10‐point increments. This produced continuous scores ranging from 0 to 100 with higher scores reflecting higher levels of COVID‐19‐related anxiety.

#### Generalized anxiety

Symptoms of GAD were measured using the Generalized Anxiety Disorder 7‐item Scale (GAD‐7: Spitzer *et al*., 2006); participants were categorized as screening positive for GAD if they scored above the cut‐off score of ≥10. The GAD‐7 has been shown to produce reliable and valid scores in community studies, and the reliability in the current sample was high (α = .94).

#### Somatic symptoms

Somatic symptoms were measured using the Patient Health Questionnaire (PHQ‐15: Kroenke *et al*., 2002). The PHQ‐15 is a 15‐item self‐report measure that asks participants the degree to which they have been bothered by physical health problems in the last 2 weeks (items are rated on a 3‐point Likert scale ranging from 0 [not bothered at all] to 2 [bothered a lot]). The scale was scored using the multi‐dimensional approach proposed by Cano‐García *et al*. (2020); following these authors, we excluded the ‘menstrual problems’ item, which contains gender‐specific content, and the ‘fainting spells’ item, which has previously been found to have low endorsement rates. The total scale scores and subscales reflecting pain symptoms, gastrointestinal symptoms, cardiopulmonary symptoms, and fatigue symptoms were used. Multiple previous studies attest to the reliability and validity of the PHQ‐15 (Hinz *et al*., 2017).

### Analysis

Scores on the COVID‐19 anxiety variable were categorized into quintiles, and the quintiles were dummy‐coded with the lowest quintile used as the reference category. This allowed to test for non‐linear associations. Regression models were used to estimate the relationship between the COVID‐19 anxiety quintiles and the PHQ‐15 subscales (pain, gastrointestinal, cardiopulmonary, and fatigue). Model 1 included the four dummy‐coded COVID‐19 anxiety variables as predictors of the four PHQ‐15 subscales; the four subscale scores were included in a single model. The regression coefficient for each dummy‐coded variable is interpreted as the difference in means between that quintile and the lowest quintile. Model 1 was also run separately with the total PHQ‐15 summed scale score replacing the subscales. Model 2 included the covariates (age, gender, income, pre‐existing health problems, and GAD) as predictors, and this model was estimated with the subscales as dependent variables and with the total scale score as the outcome variable. All four models were specified and estimated using Mplus 8.1 (Muthén, & Muthén, 2018) using robust maximum likelihood.

## Results

Descriptive statistics for all the study variables are presented in Table 1. The income bands were approximately equal (£0 – 15,490 per year [*n* = 410, 20.2%]; £15,491 – £25,340 per year [*n* = 410, 20.2%]; £25,341 – £38,740 per year [*n* = 385, 19.0%]; £38,741 – £57,930 per year [*n* = 410, 20.2%]; and £57,931 or more per year [*n* = 410, 20.2%]).

**Table 1:** Descriptive statistics and correlations for all study variables

	Age	Gender (Female)	Income	Health Problem	GAD‐7 ≥ 10	COVID‐19 Anxiety	PHQ: Pain	PHQ: Gastro	PHQ: Cardio	PHQ: Fatigue	PHQ total
Gender (female)	−.261***										
Income	.068**	−.158***									
Health problem	.112**	−.040	−.072*								
GAD‐7 ≥ 10	−.237***	.104***	−.119***	.103**							
COVID_19 Anxiety	.064**	.113***	.053*	.085*	.200***						
PHQ: Pain	−.105***	.023	−.105***	.127***	.298***	.112***					
PHQ: Gastro	−.242***	.097***	−.090***	.131***	.365***	.129***	.633***				
PHQ: Cardio	−.197***	−.069*	−.050*	.135***	.340***	.072**	.642***	.704***			
PHQ: Fatigue	−.143***	.137***	−.088***	.139***	.384***	.166***	.553***	.614***	.495***		
PHQ total	−.210***	.055*	−.098***	.155***	.411***	.140***	.835***	.896***	.853***	.770***	
Mean/*N* (%)	45.446	1,047		347	438	67.724	.942	1.140	.634	1.001	3.718
*SD*/(%)	15.901	(51.9%)		(17.1%)	(21.6%)	24.596	1.337	1.693	1.431	1.182	4.767

Note

* *p* < .05; **p < .01; ***p < .001.

COVID‐19 anxiety was positively correlated with all of the somatic symptom subscales and the total scale score. The somatic symptom subscales were all highly and positively correlated with each other, and the correlations with GAD were higher than for the COVID‐19 anxiety scores (see Table 1). Table 2 shows the estimates from the regression models. A significant proportion of variance was explained in each of the somatic symptom subscales with the exception of cardiopulmonary symptoms. A similar pattern of results emerged across all subscales and the total scale score: The effects for quintiles 2 to 4 were significant, but the magnitude was small compared to the effects for the 5th quintile. Rather than a linear increase, there appears to be an increase in quintiles 2, 3, and 4 compared to the first quintile, and then a much larger effect for the 5th quintile. For Model 2 that included the control variables, the pattern of results for COVID‐19 anxiety was similar albeit the magnitude of the effects was, expectedly, attenuated. The estimates for the 5th quintile all remained statistically significant. The effects for GAD‐7 were all large, positive, and statistically significant.

**Table 2:** Unstandardized regression coefficients from models predicting PHQ‐15 Scale and Subscale Scores

	Pain	Gastro	Cardio	Fatigue	Total scale
*Model 1*					
COVID‐19 anxiety					
Quintile 1	–	–	–	–	–
Quintile 2	0.140	0.339**	0.231	0.178*	0.889**
Quintile 3	0.189*	0.340**	0.119	0.281***	0.931**
Quintile 4	0.181*	0.289**	0.064	0.267**	0.801*
Quintile 5	0.427***	0.594***	0.251*	0.590***	1.863***
*R* ^2^	.011*	.012**	.004	.027***	.015**
*Model 2*					
COVID‐19 anxiety					
Quintile 1	–	–	–	–	–
Quintile 2	0.103	0.249*	0.164	0.122	0.637*
Quintile 3	0.141	0.251*	0.058	0.196**	0.647*
Quintile 4	0.121	0.230*	0.014	0.179*	0.544
Quintile 5	0.232*	0.327**	0.058	0.337***	0.954**
Control variables					
Age	−0.004*	−0.019***	−0.013***	−0.005**	−0.041***
Gender (female)	−0.074	0.021	−0.305***	0.123*	−0.234
Income	−0.065**	−0.052*	−0.022	−0.028	−0.167*
Health problem	0.262**	0.395***	0.360***	0.238**	1.255***
GAD‐7 ≥ 10	0.858***	1.243***	1.059***	0.971***	4.131***
*R* ^2^	.103***	.172***	.152***	.169***	.198***

Note

* *p* < .05; **p < .01; ***p < .001.

## Discussion

This study sought to determine whether self‐reported COVID‐19 anxiety levels were associated with increased somatic symptoms within a representative sample of the UK adult population. Our findings indicated that having COVID‐19 anxiety scores in the 2nd or higher quintile was associated with increased somatic symptoms, suggesting a threshold above which the chances of experiencing somatic symptoms are greatly enhanced. When adjusted for all other covariates, the highest quintile of COVID‐19 anxiety remained significantly and strongly associated with all somatic symptoms, except cardiopulmonary symptoms. The strongest association was with the fatigue subscale of the PHQ‐15.

That the effect for COVID‐19 anxiety remained even when controlling for screening positive for GAD is an important discovery because it suggests that the anxiety that people are experiencing because of this pandemic is a unique contributor to the somatic problems that they are suffering from. There is, therefore, potential for additional strain on health service providers if individuals seek help for these complaints. A public health message that explains to the public that the stress, worry, and anxiety they are feeling about the current pandemic can manifest in increased levels of physical/somatic problems, particularly fatigue, pain, and gastrointestinal discomfort, may be helpful in alleviating unnecessary worry among the public and reducing strain on the health system during this crisis situation. Of course, any such public health message must also stress that individuals with pre‐existing health problems, particularly those experiencing cardiopulmonary problems as these symptoms were unrelated to COVID‐19 anxiety and may be related to other life‐threatening conditions, should seek appropriate medical care if their physiological symptoms change in any way. Furthermore, these findings suggest there may be value in implementing psychological measures of COVID‐19 anxiety in primary care facilities for those presenting with non‐specific and possibly psychosomatic problems.

This study had some limitations. First, our measure of COVID‐19 anxiety has not been previously validated. Second, the temporal ordering of the variables cannot be established, and so the potential problem of reverse causation cannot be ignored. Third, the PHQ‐15 subscales include a relatively small number of items, and this limits the variability of the scale scores. This may have resulted in attenuated correlations and regression coefficients.

## Conflicts of interest

All authors declare no conflict of interest.

## Author contribution

Mark Shevlin, Emma Nolan (Writing – original draft), Marcin Owczarek (Writing – original draft) Orla McBride (Methodology; Writing – review & editing) Jamie Murphy (Conceptualization; Writing – review & editing) Jilly Gibson Miller (Conceptualization; Writing – review & editing) Todd K. Hartman (Conceptualization; Methodology; Writing – review & editing) Liat Levita (Conceptualization; Writing – review & editing) Liam Mason (Conceptualization; Investigation; Writing – review & editing) Anton P. Martinez (Conceptualization; Project administration; Writing – original draft) Ryan McKay (Conceptualization; Methodology; Writing – review & editing) Thomas VA Stocks (Conceptualization; Writing – review & editing) Kate M Bennett (Conceptualization; Funding acquisition; Writing – review & editing) Philip Hyland (Conceptualization; Data curation; Methodology; Writing – original draft; Writing – review & editing) Richard Bentall (Conceptualization; Investigation; Methodology; Project administration; Writing – original draft; Writing – review & editing).

## Data Availability

The data sets generated during and/or analysed during the current study will be archived with the UK Data Service (https://ukdataservice.ac.uk/) within 6 months of the study ending.
